# Structural and Practical Departments

**Published:** 1907-08-17

**Authors:** 


### STRUCTURAL AND PRACTICAL DEPARTMENTS.

### TUBERCULOUS PATIENTS IN COTTAGE HOSPITALS.

A correspondent writes :—

May I trespass on your time to ask a question connected with the local hospital here—one of about fourteen beds and three or four cots in a separate ward.

I notice in "Cottage Hospitals," 1896 edition, that phthisis is not admitted into most cottage hospitals. May I ask you is there any objection to admitting early cases of consumption one at a time, supposing the committee sanctioned the idea, and that proper precautions were taken with the sputum, etc.?

[It is clear to us that it is not justifiable to treat cases of tuberculosis in a ward with patients who are not suffering from this disease. Cases of tuberculosis must be isolated and treated apart, and it is not justifiable for hospital authorities to mix them up with other patients. The authorities of lunatic asylums, for instance, are building tubercular wards apart from the main building, and are moving all phthisical cases from the asylums into the special wards. The effect of this in the case of one asylum which we inspected recently will be, estimated from the probable reduction in the mortality of the patients, to increase the cost to the ratepayers, by £146,000. If it is justifiable to expend all this money to preserve and prolong the life of the unfortunates of our race who lack mental strength to maintain themselves, it is obvious that neither public nor scientific opinion will approve the risk of spreading infection by the reception of tuberculous patients in any public hospital, whether this be large or small.—Ed. The Hospital.]

